# Science teachers’ mentoring support experiences when integrating technology in design-based learning STEM activities

**DOI:** 10.1371/journal.pone.0332047

**Published:** 2025-09-29

**Authors:** Esra Bozkurt Altan, Tuğra Karademir Coşkun, Nurhan Öztürk, Yasemin Hacıoğlu

**Affiliations:** 1 Science Education, Faculty of Education, Sinop University, Sinop, Türkiye; 2 Computer Education, Faculty of Education, Sinop University, Sinop, Türkiye; 3 Science Education, Faculty of Education, Giresun University, Giresun, Türkiye; MNNIT Allahabad: Motilal Nehru National Institute of Technology, INDIA

## Abstract

In this study, the experiences of science teachers who applied the design-based learning (DBL) method in science lessons and received mentoring support towards technology integration were analyzed. Using a nested mixed design, the study included 43 science teachers who participated in a professional development program carried out with a mentoring model in a national project. In the study, face-to-face and online training were given to teachers, and mentoring was provided. Lesson plans and technology integration were evaluated with an online form. It was observed that teachers mainly tried to integrate technology into DBL activities but not as a component of the design process. The teachers integrated technology when teaching DBL STEM activities by using new teaching methods that incorporated instructional technologies. They carried out the integration, especially in the stages of solution development, prototyping/testing, and communication. Teachers who believed that technology supported their professional development recommended improving professional development programs to help overcome the challenges of technology integration.

## Introduction

Failure to keep up with Industry 5.0 may lead to delays in the adoption of new technologies, obsolescence of production processes, and a decrease in the ability to compete in the global market [1]. This situation has made it essential to train qualified individuals who can adapt to technology-human interaction and contribute to the transformation it brings [2]. These individuals can only be trained through technology-integrated education. In response to these needs, STEM (Science, Technology, Engineering, Mathematics) education is a modern learning approach that emphasizes the integration of disciplines and the problem-solving process [3–5]. In the STEM approach, students are confronted with a real-life problem, and in the process of solving this problem, it is ensured that they acquire the knowledge and skills of more than one discipline targeted to be taught by operating the process of multiple disciplines similar to the professionals of those disciplines [6–9]. The knowledge and skills gained through STEM education can help alleviate concerns related to Industry 5.0 [2], re-authorization of the workforce and elimination of the lack of training processes can ensure the adaptation of both individuals and societies to technological transformation.

As a method of implementing the STEM approach, design-based learning (DBL), which is based on structuring science courses around the engineering design process [7], is frequently encountered. DBL is the process of learning the necessary science and mathematics while designing a solution to a problem in a real-life context by using the engineering design process [10]. DBL encourages learning the subjects of the disciplines while developing a solution to a problem in a real-world context, and it provides the development of advanced life skills, professional skills, and competences expected to have as a businessperson in the future industry 5.0 focus [11–14]. For this reason, it has been at the centre of science education research in many countries in recent years; United States [15], the United Kingdom [16], Japan [17], Taiwan [18], and Saudi Arabia [19]. Although DBL offers opportunities in terms of science education, there are various limitations in its implementation. Teachers experience various difficulties in designing, implementing, and assessing problems in real-world contexts that are aligned with the learning objectives for DBL [20]. For example, teachers who teach with DBL prefer mechanical physics subjects in which the design process is easy to operate, while they rarely prefer it in teaching space and earth science subjects [21]. This is because designing in these areas requires a deeper understanding of science and technology. However, integrating technology into the DBL process makes teaching more challenging, as it requires greater technological-pedagogical skills, more equipment and materials, and additional time [22–26]. Unfortunately, the debates on whether technology should be considered as a discipline rather than just a teaching tool or how it should be integrated [27–29] also strengthen this difficulty. In the integration of technology in STEM, technology should be an active component of the solution process, not a tool (only instructional technology) [29–32]. On the other hand, it is known that science teachers often cannot go beyond using technology as a tool in learning environments suitable for the STEM approach [33,34]. Similarly, in technology-focused STEM activities implemented in primary and secondary schools between 2015 and 2023, technology has generally remained at the level of instructional technology as a tool for teaching science and mathematics [32]. For this reason, it is essential to support teachers’ professional development on how to integrate technology within the DBL method as a way of implementing the STEM approach and to provide mentoring by field experts to further assist them. The positive relationship between STEM teachers’ digital literacy and their integration of technology discipline into learning and teaching processes [35] reveals the need to support teachers in this regard. Within the scope of this research, a professional development program based on a hybrid mentoring model was prepared to support science teachers’ competence in applying the DBL method in their lessons and preparing lesson plans using the DBL method. The focus of the research is on how teachers participating in this program integrated technology into DBL activities. Supporting teachers to integrate technology into their lessons and examining the integration processes in detail will provide important insights into how technology integration can be optimized in the DBL context. Determining the best practices for the problems arising in this process will be guiding for the literature. In this context, the study aimed to investigate how science teachers, who participated in a national project with mentoring support for using the DBL method in science lessons, integrated technology into their DBL lesson plans and to explore their experiences.

For this purpose, the following questions have been addressed:

1. In DBL lesson plans, how and at which stages do teachers integrate technology?
2. How did technology integration processes during DBL activities affect teachers’ professional development?
3. From the teachers’ perspective, what are the effects of technology integration processes in DBL activities on students’ skill acquisition?

## Theoretical background

### STEM education and design based learning (DBL)

STEM is a learning approach based on the holistic teaching of two or more of the disciplines of science, technology, engineering, and mathematics in the context of real-life problems [7,9,36,37]. There are many methods on how science and other STEM disciplines should be integrated while performing STEM education. The most common methods are problem-based learning in STEM, project-based learning in STEM, and DBL. Unlike problem-based learning and project-based learning, DBL, where the engineering design process plays a central and highly effective role [7,38,39]. DBL is based on structuring science courses around the engineering/technology design process [7]. Since the DBL method directly integrates engineering into the lessons, it stands out from other methods as a good way to implement the STEM approach in the classroom [12]. DBL is learning method that combines the scientific research process and the engineering design process to solve real-life problems related to STEM disciplines. Within this framework, students acquire knowledge from relevant fields and apply it in the design process to develop practical skills. Therefore, it is also referred to as classroom practices of engineering based on real life [10]. DBL bridges the gap between classroom learning and the real world by facilitating the integration of theoretical knowledge with practical application [13]. This enables students to understand the scientific concepts behind design and equips them with the practical skills necessary for careers in fields such as engineering and technology [12]. Students gain rich experience because they are required to actively participate in the process [40]. In the DBL process, students conduct scientific research while designing solutions to real-life problems and work in communication and cooperation with a critical perspective. This process supports students in developing skills aligned with the demands of the 21st-century workforce by encouraging creative and productive solutions. In addition, the operation of the iterative design process encourages the development of growth mindsets through feedback and reflection provided to the students [12,41]. The operation of the iterative design process develops resilience and adaptability skills, which are increasingly important in a fast-changing labor market. Moreover, DBL enhances students’ teamwork skills and prepares them for collaborative work environments as many projects require collaboration among peers [40,42]. The focus on creating concrete solutions/outcomes with DBL consolidates the understanding of abstract concepts and makes learning more meaningful and effective [12]. In this study, the DBL method [43,44] was adopted as a way to apply the STEM approach to strengthen science lessons in interdisciplinary contexts. For this reason, the focus was on science lessons.

### Integration of technology to STEM education and DBL

Integrating technology into STEM education is important and necessary to achieve the goals required by the age. However, to determine how to integrate technology into STEM education, the nature of this discipline needs to be understood. The definition of technology as ‘what is done to meet people’s wants and needs’ [45] points out that technology integration should be more than the use of instructional technology as a tool [27,29] and that technology should be a design-based process involving the application of science and mathematics knowledge [29,46]. However, in practices that will improve technological literacy, such as the use of instructional technology [47], the use of particle accelerators obtained from sensors, software, microscopes, and supercomputers used to collect data [27,48], planning applications to develop algorithmic thinking skills [49,50], the implementation of robotic applications [51], the use of information and communication technologies [52], the creation of digital stories, creating projects using chrome books and google services, website creation, designing brochures [53], maker tools [32], the use of information and communication technologies are preferred [32]. All of these practices may support technological literacy in some way, but when considering the aims and objectives of STEM education, technology integration must go beyond the use of instructional technology as a tool [27,29]. It is important, and even necessary, that DBL activities involve a design-based process that includes the application of science and mathematics knowledge [29]. In support of this conclusion, research on technology integration in STEM education indicate that despite criticisms, students’ learning experiences and outcomes can be improved even in this way [54,55]. The fact that technology should go beyond being a tool and that science and mathematics knowledge should be handled in an applied process can also be a solution to the discussions on whether it should include information and communication technologies (ICTs), engineering technologies, STEM hardware tools, producer tools, plugging technologies, plugging non-plugging technologies, supportive teaching technologies or integrated coding technologies [30,31,53,56]. In light of discussions in the literature, this study evaluates a specific application in terms of technology integration, emphasizing that in the context of STEM, an essential criterion is the use of technology as an active component in the problem-solving process, rather than merely as a tool. In other words, it is important not only to support the use of technology in STEM teaching but also to engage students in designing and producing technology. In this context, it is considered that the use of technology in a way that enables it to produce solutions to design problems and facilitate student interaction is an essential component [57]. On the other hand, it is thought that the use of technology as a part of the scientific research process in the DBL process can also be considered as technology integration to some extent. For example, a scientific research inquiry process that contributes to the design of a product with simulations can also be considered as technology integration. However, it is considered that the use of technology solely as a teaching tool is not compatible with the paradigm of the STEM approach. It would be more meaningful for the technology discipline to provide the integration of theory and practice, which is one of the basic principles of the STEM approach and DBL, which is a method suitable for this approach.

With its innovative structure, DBL facilitates technology integration, enabling students to design solutions to real-world problems [58] and ensuring that they solve problems through technology design by using scientific knowledge from a multidisciplinary perspective [44]. In addition, students gain the ability to use their knowledge and skills through technological design, establish a relationship between subject matter and design tasks, develop a multidisciplinary perspective, and develop a positive attitude toward learning [59,60]. Technology integration with the innovative structure of DBL facilitates students to design solutions for real-world problems. Technology integration in DBL activities, for example, enables the integration of digital tools and multimedia resources and enables interactive and engaging learning activities [61,62]. This process supports DBL’s goal of developing skills such as problem-solving and critical thinking by encouraging students to develop creative solutions to real-world problems.

### Science teachers’ technology integration to STEM and DBL

Teachers play an important role in the effective integration of technology into STEM learning-teaching processes. Indeed, teachers are important for the effective implementation, adoption, and increase of pedagogical applications such as technological DBL, as well as for overcoming the difficulties encountered in their implementation [57]. Integrated STEM teachers have indicated that various factors influence successful STEM implementation, including pedagogical knowledge of subject-discipline integration, collaboration with other teachers in implementation, and mentoring [63,64].

Teachers’ efficacies in integrating technology into science, mathematics, and engineering subjects predict their efficacy of integrative STEM teaching [65]. In the limited research on how technology integration is done by teachers in the STEM approach, it has been determined that science teachers primarily use technology (along with mathematics) as a tool or way to support science and engineering activities in their classrooms [34]. Technology has been utilized in the form of smart boards for teaching and videos to improve students’ understanding of science content, as well as software to help students plot graphs of data collected in a science project.

Teachers have difficulty in verbally defining what technology means in the STEM approach and sometimes even refer to technology as a ‘mysterious part’ [34,66]. Teachers who try to use technology/engineering DBL approaches in their lessons generally avoid technology DBL applications because they are not sufficiently familiar with the DBL process and technological design, programming, electronics, etc., which enable the integration of technology. They have also noted that they are unable to establish interdisciplinary connections with science subjects and carry out collaborative interdisciplinary work, leading them to avoid technology DBL applications [57,63].

Teachers’ concerns and uncertainties regarding the integration of technology into STEM activities also indicate their perception that there should be a difference from traditional teaching technologies [34,66]. On the other hand, the technology dimension of STEM is generally associated with engineering processes [66]. Taking a specific and in-depth look at how technology is integrated into the STEM approach within design-based learning (DBL) is a unique contribution, and this need is also highlighted in the literature [67]. It is well known that practical studies on supporting teachers for successful technology integration will contribute significantly to the field.

### Hybrid mentoring model for teachers’ professional development

The ultimate goal of professional development activities is to enrich teachers’ classroom practices and improve student achievement. This goal is a high expectation. Theoretically, it will be necessary to enrich the teacher’s knowledge and skills and to change their beliefs and behaviors related to their field of activity [68–70]. The literature highlights the effectiveness of mentoring practices in teachers’ professional development processes for sustainable professional development [71–74]. Mentoring can be defined as the process by which an experienced expert supports the professional development of a less experienced individual by sharing knowledge and experience within a relationship of mutual trust [75–77]. Researchers emphasize that the professional development needs of teachers who continue in their profession should be met through a mentoring model in order to improve their pedagogical knowledge [71,78]. The literature also includes studies on the professional development of teachers who continue in their profession in various areas through mentoring [71,79–84]. The study found that teachers reported improvements in many skills during their professional development process through mentoring, including planning and implementing lessons in line with active learning strategies, conducting assessments and evaluations, planning homework, developing daily writing skills, and establishing effective communication with students [82]. Limited research conducted to improve teachers’ competence in integrating technology into STEM activities also highlights the need for professional development programs in this area. The application of the technological pedagogical content knowledge framework has been effective in helping mathematics teachers integrate their technology, pedagogical, and subject knowledge to support sustainable professional development in STEM fields [85]. Teachers from various disciplines who participate in a short-term technology-supported STEM professional development program may experience an increase in perceived ease of use, perceived benefits, attitudes toward technology, and intentions to use technology [86]. Applied research findings show that both the integration of technology into STEM activities [85,86] and the use of contextual, school-based, and community-focused approaches play a critical role in ensuring the sustainable impact of STEM professional development programs [67,87]. Additionally, the importance of school leadership and peer support in shaping teachers’ attitudes and practices toward technology integration is emphasized [57,85].

## Method

### Research design

This study was designed to examine how teachers realize technology integration into DBL lesson plans and their experiences. The preference for an nested sampling in mixed methods in this study is related to the broad scope of the research and the need for in-depth explanations that contextualize the research findings [88]. The reason for choosing this pattern is that technology integration in the DBL context is a multi-layered phenomenon. Dimensions such as teachers’ technical skills, pedagogical practices, and emotional responses can be evaluated more comprehensively using information obtained from both quantitative and qualitative data sources [89]. In this study, the quantitative data obtained during the examination of DBL lesson plans were reported with descriptive analysis techniques in order to determine teachers” technology integration preferences, the stages of this integration, and their general tendencies. However, it was recognized that these quantitative findings alone may be insufficient to explain contextual dynamics. For example, explaining the reasons underlying the intensive use of technology at a certain stage or the changes in teachers’ emotional states necessitated the need for qualitative data. Hence, the interview data collected from the teachers were evaluated with qualitative analysis techniques and structured in a way to support the numerical findings. The findings obtained from quantitative data are not generalizable because no inferential statistical analysis was performed. Instead, when considered together with qualitative data, they have the power to explain meaningful patterns and trends specific to the context of the study. In this study, quantitative data were used to reveal teachers’ general tendencies and approaches towards the stages of technology integration, while qualitative data were used to explain teachers’ individual experiences and emotional reactions during these processes. Therefore, this study aims to provide transferable conclusions for practitioners in similar teaching contexts rather than statistical generalizations. The findings are particularly relevant for guiding decision-making processes regarding technology integration in DBL activities.

### Participants of the research

The study group of this research consists of 43 science teachers who received training on planning science lessons and technology integration with the DBL method within the scope of a project carried out with a mentoring model at a national level. Participants are middle school [grades 5–8] science teachers. Participants were selected based on the criterion of being teachers working at general and official boarding secondary schools in the regional centers of the country where the project was carried out and at Science and Art Centers where gifted students receive education. The working group was determined in two stages. In the first stage, the maximum diversity sampling method was used in the purposeful sampling method to determine the working group. This method, which is one of the types of purposive sampling, aims to identify and define main themes that include many different characteristics [90]. In line with this, the aim was to ensure diversity in the selection of the working group by selecting teachers from boarding regional secondary schools, Science and Art Centers, and general secondary schools. The participants of the study work in various contexts such as Science and Art Centers (n = 6), general secondary schools (n = 33), and low socioeconomic regional boarding secondary schools (n = 4). This approach was chosen to make the study’s results more generalizable [91] in a way that ensures applicability not only to one context but also to a wide range of educational systems. This diversity is particularly important given the suitability of the DBL method for context-sensitive pedagogical practices. In order to ensure regional representation, one boarding secondary school, one Science and Arts Center, and four teachers working at two general secondary schools from each regional center participated in the project, which was carried out with the participation of 48 teachers. Increasing regional representation is another criterion of the research. This is because regional diversity provides theoretical and practical insights into how education is implemented in different contexts. Rogers’ Diffusion of Innovations Theory [91] emphasizes that the local context plays a critical role in the adoption process of innovative practices and technologies. Within this framework, participants were selected to represent each of the seven geographical regions of the country.

The link to the project website and promotional posters have been published on platforms such as Provincial and District National Education Directorates, social media accounts of project personnel, and social media teacher groups. A period of four weeks has been allocated for teachers to apply. Applications have been classified according to the criteria specified above. In the second phase, a certain number of teachers were selected from among the applications submitted by teachers working in general and boarding middle schools and Science and Art Centers in each province and in those provinces through simple random sampling. Thus, teachers who met the diversity criteria specified in the first phase were given an equal chance.

The participants in this study were classified according to their professional seniority years as follows: 7 teachers with 1–5 years of seniority, 10 teachers with 6–10 years of seniority, 15 teachers with 11–15 years of seniority, and 11 teachers with more than 16 years of seniority. Participants also show diversity in terms of education levels. While 20 of the participants have only undergraduate education, 6 of them continue their postgraduate education. Additionally, 13 participants have completed their master’s degrees, 3 participants are currently pursuing their doctoral degrees, and 1 participant holds a doctoral degree. This enables a broader perspective on the effects of both professional experience and academic education level on the integration of technology into the DBL method.

### Data collection tools

**Fig 1:**
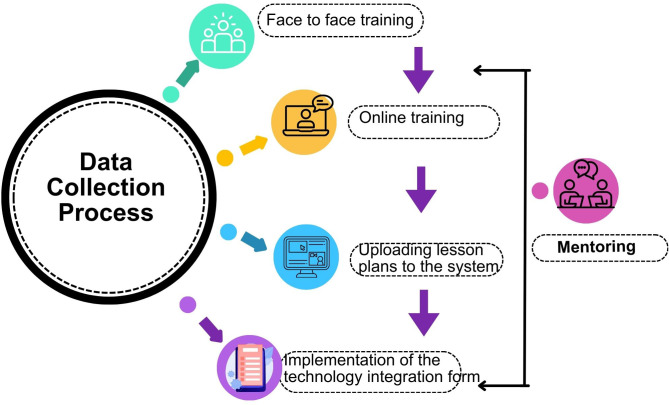
Data collection process stages.

The data for this study were collected as part of a national project that aimed to develop a hybrid mentoring-based professional development model to enhance science teachers’ competencies in implementing design-based learning (DBL) activities in the classroom. In this context, after the teachers were identified, the data collection process was carried out with the stages presented in Fig 1:

The data collection process stages presented in Fig 1 also represent the structure of the professional development program.

The data collection process stages presented in Fig 1 also represent the structure of the professional development program. Within the scope of the professional development program, teachers were first provided with face-to-face training, and at the end of the program, a mentor was assigned to every 3–4 teachers to initiate the mentoring process. Mentors held regular meetings with mentees and provided support to them to improve their competences in preparing and implementing lesson plans in accordance with the DBL method. The content of the online training was determined based on the deficiencies identified during this process. Unlike the mentoring process, the online training provided all participants in the professional development program with training on the content identified by mentors as necessary for the classroom application of the DBL method. Teachers received feedback from mentors on the lesson plans they began preparing during the initial face-to-face training, and their plans were finalized after the online training.

***Face-to-face Training Phase:*** The data collection process of the research started with face-to-face training for teachers. The training program was carried out with an accommodation structure lasting 6 days and consisting of a total of 55 lesson hours. In the general framework of the training, the STEM approach and its importance, DBL method and its stages, and applications related to the DBL method were included. The training was conducted by 11 academicians specializing in science education, technology, and engineering. At this stage, it was aimed for the teachers to understand the DBL method and STEM approach and the importance of these methods in learning processes. At the same time, teachers were given the opportunity to analyze lesson plans based on DBL and gain theoretical knowledge about these plans. Following this general framework for DBL, examples of various technology DBL activities with teachers were presented [92], and the integration of technology into science education through DBL was elaborated. In addition, participants were given practical and applied training to support technology integration into the DBL process. On that note, the topics given to teachers in practical training are as follows:

- Robotic Applications: Teachers were informed about how robotic systems can be used in lessons with sample applications. In this context, Arduino training was given.
- Animation Preparation: Animation software is introduced to prepare creative and interactive animations. Powtoon, one of the animation preparation software, is focused intensively on the application.
- Simulations: Interactive simulations that can be used in science lessons were introduced to teachers. Platforms such as Phet Colorado, Circuit Board, and Thinkercad that support students’ learning by experimenting especially in physics, chemistry, and biology subjects were presented to teachers.
- Digital Concept Map: It was introduced as a method that teachers can use to make sense of the relationships between concepts. In this context, the use of Cretaly and Canva was focused.
- Digital Interactive Game Platforms: The platforms used to create interactive games and quizzes were introduced to teachers. These tools are enriched with applications that aim to increase students’ active participation and make learning fun. Wordwall and Learning App were shown in this context.
- E-Portfolio: Systems for creating an e-portfolio and their use are described.
- Poster/ Infographic creation: The visual design tool Canva was introduced to teachers to create lesson materials, posters, and infographics.
- Augmented Reality (AR) Applications: Augmented reality technologies have been introduced to enable students to engage with learning materials more interactively.

**Fig 2:**
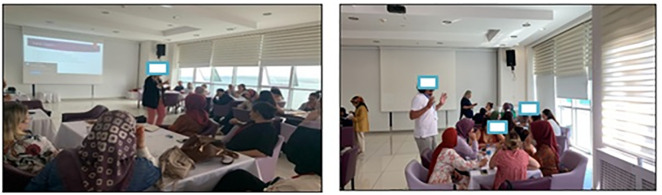
Examples of face-to-face training.

During the training process, teachers were provided with examples of how all these technologies should be integrated into the DBL process and what kind of DBL activities they can design and implement so that they do not remain as instructional technology. In addition, a template for preparing lesson plans in accordance with the DBL method was introduced and they were provided to develop draft lesson plans in line with this template. The drafts were analyzed by other participants and experts, and individual feedback was provided to each teacher. Teachers had the opportunity to improve their lesson plans in line with this feedback. Visuals from the face-to-face training process are presented in Fig 2.

Fig 2 shows examples of sessions from the face-to-face training process conducted as part of the study.

This process provided teachers with a deeper understanding of the DBL method and increased their knowledge and skills in technology integration. The training provided a comprehensive structure for teachers to develop both their theoretical knowledge and practical application.

**Fig 3:**
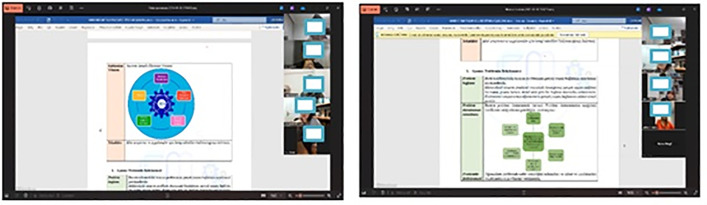
Images from online meetings about the mentoring process.

***Mentoring Process:*** Each of the experts on DBL in the project team was assigned as a mentor to 3–4 teachers (mentees). Mentors held regular meetings with mentees and provided support to them to improve their competences in preparing and implementing lesson plans in accordance with the DBL method. Mentors and teachers held regular meetings every week. The mentors gave feedback on the lesson plans, guided the teachers in implementing the finalized plans in the classroom, and encouraged them to share their implementation experiences. At this stage, the mentoring process continued for 8 weeks. Meetings were held every week for 40–90 minutes. The records of the mentor and mentee meetings, the activity materials used during the face-to-face training in the project, and all materials that would contribute to the integration of technology, engineering, and mathematics disciplines into the activities of the students were stored in a learning management system where teachers and mentors were registered. The learning management system was also prepared within the scope of the project. Teachers were able to review the records at any time, ask questions to the mentors through the system, and receive feedback. Visuals of the meetings held during the mentoring process are presented in Fig 3.

Fig 3 presents images from meetings held with teachers during the online mentoring process.

**Fig 4:**
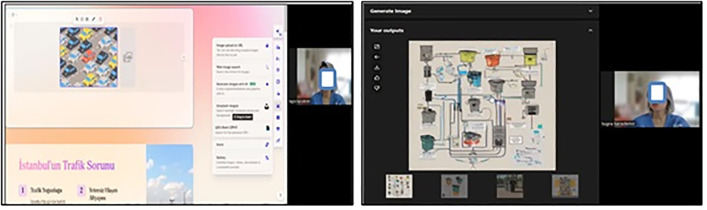
Online training images.

***Online Training:*** At this stage, a total of 25 hours of online training was conducted. In the training, topics such as lesson plan preparation and implementation experience sharing, tools that can be used for technology integration into the DBL method, the importance and how to do pre-assessment, process and summative assessment practices, organizing activity sheets, and lesson plan preparation were discussed. After this training, all teachers reviewed and finalized their lesson plans. Online training visuals are presented in Fig 4.

Fig 4 shows images of the content and participant interactions used in online training sessions.

In the online training phase, teachers were introduced to a set of generative AI tools for technology integration to add value to lesson plans and improve teaching processes. During the introduction and use of these tools, practical trainings were included. The introduced artificial intelligence tools are:

- ChatGPT- Gemini: It was used to guide teachers in areas such as creating lesson content, problem-solving, developing activity suggestions, and writing texts.
- Gamma: It was introduced to teachers as a tool for preparing effective and visually rich presentations and taught ways to improve the visual quality of educational materials.
- Speech Synthesis: It was used to add voice-overs to educational materials and to increase student interactions.
- Elicit and Scite: It was introduced to assist teachers in the process of searching and referencing sources. These tools enabled teachers to find and organize scientific resources more effectively.
- InVideo: This tool, which is used to prepare video content quickly and professionally, was introduced specifically to increase the use of audiovisual material in courses.
- YouTube Instant Language Translation Tools: This tool enables teachers to use videos presented in different languages, creating an inclusive learning environment for students.
- Concept Map Preparation Tools with Prompt: By generating concept maps automatically, these tools helped teachers save time and visualize the course content.
- Software for Turning Videos into Blogs: It was used to facilitate student access by converting learning materials into different formats.

These tools were intensively transferred to teachers for the design of instructional materials and improvement of lesson processes in accordance with the DBL method. During the training process, teachers had the opportunity to experience different dimensions of technology integration by making applications with these tools.

**Fig 5:**
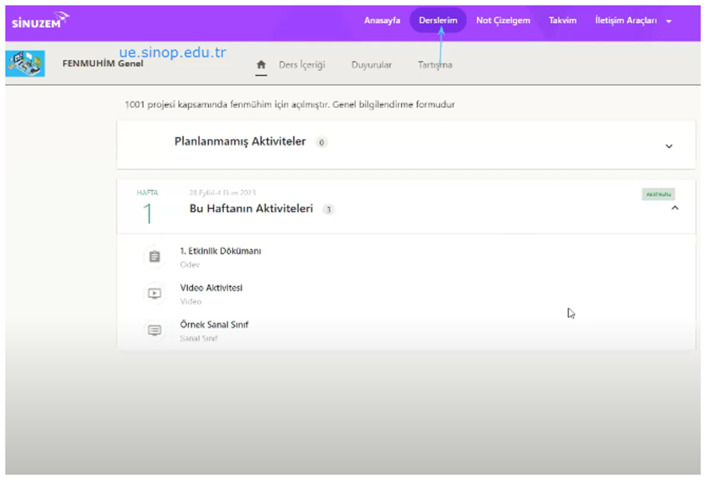
Lesson plan images uploaded to the learning management system.

***Teachers uploading lesson plans to the Learning Management System:*** Following the online training, a mentoring process was initiated through the learning management system (LMS) for teachers to finalize and upload the final versions of their lesson plans. This process was structured to enable the teachers to perform quality control and finalization of their lesson plans. Teachers were given a total of 4 weeks to upload the final version of their lesson plans. In this process, some teachers prepared and uploaded more than one lesson plan. As a result, a total of 52 lesson plans were obtained at the end of the mentoring process. These lesson plans were collected through the learning management system and the files in the system were categorized in an orderly manner. The interfaces used in the learning management system are presented in Fig 5.

Fig 5 shows images of learning management system interfaces and lesson plans uploaded by teachers.

***The implementation of the Online Technology Integration Form:*** After the teachers uploaded their lesson plans to the learning management system, an online form was applied to evaluate the technology integration in these plans. This form was prepared to determine the level of technology integration in lesson plans and to measure teachers’ thoughts about technology integration. The results of the form revealed the scope and effectiveness of digital tools used in lesson plans as well as teachers’ perceptions of technology integration. This evaluation provided an important source of feedback to identify teachers’ strengths and areas for improvement in technology integration processes.

### Data collection tools

In this study, the lesson plans prepared by the teachers and the online form applied to evaluate the technology integration in these plans were used in the data collection process. Lesson plans were used as the primary data source that reveals the content developed by teachers within the framework of DBL and their practices towards technology integration. The online form was used to determine the level of technology integration in these lesson plans and the contribution of the process to teachers’ development. Details about these tools are given below:

**Lesson Plans:** Teachers prepared lesson plans suitable for DBL based on the lesson plan template developed within the project. This template guided the teachers to plan the lesson process in detail and to create activities including the basic stages of the DBL method. The related template is given in Table 1.

**Table 1:** Elements and content of the activity template.

Section	Content
Descriptive Section	Class level, learning outcomes, tools and materials, characteristics of the learning environment, prior knowledge that students should have, and the techniques used are explained.
Stage 1: Defining the Problem	In the context of the problem (1), the presentation of the problem situation (2), and the identification of the problem (3), what the teacher should do in the classroom is explained.
Stage 2: Developing Possible Solution	It is explained what will be done to determine the needs for the design problem (1). The mini research and design activities (2) planned by the teacher to develop knowledge and skills related to the problem are explained in detail. If there are any activity sheets that can be used in the classroom for mini research and designs, they are added to the lesson plan as an attachment. The actions to be taken for directing students to develop individual solution proposals (3) and for groups to come together and share their solution proposals (4) are also explained in this section.
Stage 3: Choosing the Best Solution	The steps to be taken and the tools to be used (decision matrix, decision tree, etc.) for the evaluation of the solution proposals of the groups (1) and the selection of the most appropriate solution (2) are explained here. If necessary, additional activity sheets are shared.
Stage 4: Prototyping and Testing	The instructions to be presented in the Prototype Construction (1) process, how testing will be done (2) and the situations that need improvement (3) are explained here. In this stage, the assessment rubric is additionally included.
Stage 5: Communication	Expectations and guidelines for the presentation of the designs are indicated here.
Abstract	The whole activity time is briefly explained.
Assessment and Evaluation Activities	It is explained what kind of measurement and evaluation activities are in the activity process. Additional assessment and evaluation activities can be added.

The components of the lesson plan template were created as a result of a comprehensive literature review on the DBL method. Each phase of the template has been structured based on pioneering studies in the field of DBL [7,8,10,36,39,93–99]. The lesson plan template file is provided in S1 File: DBL Lesson Plan Template. In addition, all lesson plan data has been shared openly. The template includes the basic stages of DBL such as problem definition, solution development, prototyping, testing, and communication. To ensure content validity, the template was analyzed by a group of academics who are experts in STEM education, instructional design, and DBL. The experts assessed to what extent the template reflects the DBL method pedagogically and methodologically. Improvements were made to the template in line with the feedback received. Construct validity studies were carried out to evaluate the suitability of the template for the DBL method. Each stage (e.g., problem identification, prototype construction, and testing) is described in detail to ensure its applicability within the educational process and alignment with instructional objectives. Furthermore, additional materials such as activity sheets, rubrics, and assessment tools that can be used at each stage are integrated into the template. In order to increase the reliability of the template, a panel of academics who are experts in STEM education, instructional technologies, and DBL was formed. The experts evaluated the consistency and ease of use of each component of the template. Expert opinions constituted an important source of data in terms of the applicability and functionality of the template by teachers. As a result of these studies, it was determined that the template is suitable for the DBL method and can be used as an effective tool in teachers’ lesson plan preparation processes.

**Survey on technology integration into DBL:** In the study, a questionnaire was developed to determine teachers’ evaluations of technology integration into the DBL process. This questionnaire was structured to comprehensively assess the teachers’ status of technology integration into lesson plans, at which engineering design stage they use technology, with which tools they integrate technology, teachers’ experiences with technology integration, the difficulties they encounter, the contributions they achieve and the effects of integration on students. These studies were taken as a basis for structuring the questions of the questionnaire. The questions in the questionnaire were designed to evaluate the stages of the DBL method and different dimensions of technology integration. The questions focused on key issues, including which phases of the engineering design process teachers use technology in, which tools they utilize, the challenges they encounter, and the impact of technology integration on both students and teachers. The questions were prepared in open-ended and short-answer format. The questionnaire was analyzed by the other members of the research team for relevance and inclusiveness. The experts checked whether the questionnaire was appropriate for assessing teachers’ technology integration processes and provided their feedback. In line with this feedback, the questionnaire was adjusted in terms of language and content. Reliability studies of the questionnaires are mostly based on ‘expert consensus’ or ‘inter-coder reliability’. “In the first stage, three researchers with expertise in DBL and technology integration reviewed the questionnaire items. The experts evaluated the relevance, clarity, and comprehensiveness of the questions. In this context, the questionnaire was finalized. Consisting of 15 questions, the questionnaire was applied to teachers online through short answer and open-ended questions.

### Data analysis

#### Analysis of lesson plans.

At the end of the analysis process, according to the first criterion in Table 2 (technology being an active component of the tool/solution process), lesson plans that addressed technology integration only with the ‘use as a tool’ approach were excluded because they did not meet the qualification of ‘technology being a part of the scientific research process or producing solutions to the problem’ as emphasized in the literature. Thus, more meaningful results were obtained regarding the level and quality of technology integration in supporting DBL processes. The Technology Integration Assessment Descriptive Analysis Framework, which is presented in detail in Table 2, forms the basis of the descriptive analysis conducted on the lesson plans in this study and enables the findings to be interpreted on both a valid and reliable basis.

**Table 2:** A descriptive analysis framework for evaluating technology integration in DBL activities.

Criterion	Content
Technology is an active component of the tool-solution process	Technology was used in the process of producing a solution to the design problem.
	Technology was used as a part of the scientific research process in the DBL process.
	Technology was used only as a teaching tool.
Technology Integration in the Stages of the DBL Process	Identifying the Problem
	Development of Possible Solutions
	Selection of the Most Suitable Solution
	Prototyping and Testing
	Communication
The effectiveness of technology in transforming theoretical knowledge into practice	Technology is ineffective in transforming theoretical knowledge into practice.
	Technology turns theoretical knowledge into practice to a limited extent.
	Technology effectively supports the translation of theoretical knowledge into practice.
The alignment of technology with the overall objective of the activity	The technology is not directly linked to the purpose of the event.
	The technology is partially aligned with the purpose of the event but makes a limited contribution.
	The technology clearly serves the purpose of the event.
Innovative and interactive use of technology	The technology is not innovative but remains in passive use.
	The technology is innovative, but the level of interaction is limited.
	Technology is innovative and used in interaction with students.
	Technology is used in an interactive way that is both innovative and deepens the students’ learning process.
Physical or digital prototyping and use of simulation	No use of prototypes or simulation.
	Prototypes or simulations are used to a limited extent.

In this evaluation process, two academicians who are experts in the fields of DBL and technology integration were actively involved, and an independent external evaluator also reviewed the analysis findings and provided feedback on data consistency and content validity. In accordance with the criterion of “technology being an active component of the tool/solution process” emphasized in the framework, lesson plans containing technology used only as a “tool” were excluded from the evaluation. All the data obtained were also supported by qualitative interviews and reported, allowing a comprehensive evaluation of the results of the analyses.

An example lesson plan evaluation is as follows: A problem situation was presented in a lesson plan to those who go on picnics and camping without harming nature, within the framework of designing a solar oven using solar energy. The plan included the following guideline in the technology context: “Your design model should be created using the 3D drawing program Tinkercad on a computer.” In this lesson plan, technology was used in the process of producing a solution to the design problem. Technology was integrated into the prototyping and testing stages of the DBL process. Technology effectively supported the transformation of theoretical knowledge into practice. Technology clearly served the purpose of the activity and was used in an innovative and interactive way that deepened students’ learning processes. Prototypes or simulations were used to a limited extent. Another lesson plan included a problem situation aimed at designing an insulated hood model that would minimize engine noise. The Arduino Science application was used in the lesson plan. The plan included the instruction, “In your experiment, use the Arduino Science program to measure the sound intensity and write it in a table.” This lesson plan was evaluated as being effective in supporting the conversion of theoretical knowledge into practice, serving the purpose of the activity clearly, and using technology innovatively and interactively that deepens students’ learning processes, although it was used in the prototype creation and testing phase and in the process of generating solutions to design problems. However, there was no use of prototypes or simulations.

In this study, To examine whether and how technology was integrated into the DBL lesson plans prepared by the teachers, the researchers used a descriptive analysis framework they developed for assessing technology integration. The development process of the framework consists of five stages. In the first stage, the “Objective and Scope Determination” stage, the objective of creating a framework for a comprehensive evaluation of technology integration was set in order to determine whether technology is included in DBL lesson plans and if so, in what quality. In the second stage, ‘Literature Review’, studies on technology integration in STEM/DBL activities were examined to determine which components or criteria were taken into consideration in similar studies [100,101]. In this process, the findings that the DBL approach and technology integration facilitate bridging the gap between theory and practice were taken into consideration. In the third stage, “Defining Descriptive Analysis Criteria”, the criteria to be considered in the lesson plans were determined in line with the information obtained from the literature review. These criteria presented in Table 2 include different dimensions such as the role of technology in the activity (is it only a tool, is it an active component of the solution process, etc.), how and to what extent technology is used in the stages of the DBL process (problem identification, development of possible solutions, prototyping, testing, communication, etc.), the level of innovative and interactive use of technology, the function of technology in prototype or simulation processes, and the effectiveness of technology in transforming theoretical knowledge into practice.

In the fourth stage, ‘Validity and Reliability Studies’, expert opinion was sought in order to increase the suitability of the descriptive analysis criteria and the level of serving the purpose. Two experts who have research in the fields of DBL and technology integration examined the developed framework and provided feedback on the adequacy of the criteria in terms of both theoretical and practical aspects. In addition, 10 sample lesson plans were analyzed according to the framework, and the problems or ambiguous points encountered by the researchers were revealed. In this exercise, it was identified which criteria were not clearly understood or which situations overlapped with different categories (e.g., technology merely remaining in a passive role). In line with the findings, minor revisions were made to the framework and the criteria were finalized.

In the fifth stage, “Implementation of the Framework (Inter-Coder Agreement)”, all lesson plans prepared by the teachers were analyzed by two independent researchers based on the descriptive analysis framework. Each researcher rated or described the technology integration in the lesson plans based on the criteria in Table 2. When the results of the researchers’ analyses were compared, the percentage of agreement was calculated using the formula suggested by Miles and Huberman (1994) [102] and found to be 94%. This value shows that there is a high consensus among the coders and indicates that the framework used in the evaluation of the lesson plans is quite strong in terms of reliability.

#### Analysis of survey on technology integration into DBL.

In this section, full details of the analysis stages of the open-ended and multiple-choice data collected from the teachers regarding technology integration into the DBL process are presented. The analysis process was structured in a way to allow for a multidimensional evaluation of teachers’ use of technology and their approach to DBL activities. In this context, while the quantitative data obtained from short-answer questions were analyzed by statistical methods (e.g., frequency, percentage distributions), the qualitative data obtained from open-ended questions were coded in two cycles and reported by enriching them with direct quotations to support each other. Firstly, frequency analyses were performed on the quantitative data obtained through the questionnaire; thus, the stages at which teachers used technology in their DBL lesson plans, the effects of this integration on their professional development, and the skills they provided to their students were determined. In the second stage, the qualitative data obtained from the open-ended questions were coded in two cycles. In the first cycle, the data set was scanned in general terms using open coding; at this stage, the prominent keywords and direct quotations from the teachers’ statements were determined. A detailed coding scheme was created for this process and each code was categorized under the relevant themes. In the second cycle, the axial coding phase was initiated, and the coding scheme from the first cycle was integrated with the findings from frequency analyses and examined in depth within thematic groups.

In order to reinforce the validity and reliability elements of the coding process, three researchers who are experts in the field coded together, and it was determined that the inter-coder reliability value was above 80%. In addition, an independent researcher coded the open-ended responses of the participants separately according to the same scheme; Krippendorff’s Alpha value was found to be 0.78, confirming that the reliability level of the coding process was quite good. All the findings were analyzed and interpreted holistically in a way to reveal different aspects of technology integration in the DBL process.

#### Validity, reliability, ethics.

In order to reach valid and reliable results, rigorous, systematic, and versatile methods were applied in data collection, analysis, and interpretation processes. Quantitative data were obtained through questionnaires collected from teachers. These surveys were designed to understand teachers’ general tendencies and approaches to technology integration processes. In order to ensure content validity, the survey questions were developed based on the literature and revised by taking the opinions of field experts. This process ensured the removal of the questions that were either inappropriate for the objectives of the research or misleading and formed the basis for the measurement tools in the survey to produce valid results [88]. Triangulation was applied as an effective approach to increase the validity of the results by using both quantitative and qualitative data sources. By comparing the information obtained from different data sources (questionnaires, lesson plans), the accuracy of the findings was tested. Triangulation strengthened the accuracy and reliability of the findings by balancing the limitations of one method with other methods [103].

For the analysis of qualitative data, a two-stage coding method was applied to elaborate the contextual and individual dimensions. In the first stage, raw data were classified and categorized using open coding. In the second stage, these categories were transformed into themes with the contributions of field experts, and content accuracy was evaluated. This process is a recommended approach to increase the validity of qualitative analyses [102]. This practice minimized the subjectivity that may occur between the researchers and increased reliability in data analysis. The consensus among different researchers contributed to the objective creation of the final themes [104]. Expert opinions not only deepened the research findings but also ensured the accuracy of the context. Another important factor that increased the reliability of the research was the inclusion of direct quotations in the text to support the findings obtained during qualitative data analysis. Direct statements taken from the survey findings played an important role in increasing the transparency and accuracy of the data analyses. This approach is recognized as a recommended reliability strategy in qualitative research [104]. To increase the validity and reliability of the research, the entire research process was explained in a transparent and detailed manner in the article because explaining the process in detail can provide a basis for the replicability of the research in different contexts, as suggested in the literature, by allowing readers to better evaluate the research methods and results [102]. Thus, details of the data collection, analysis, and interpretation processes were presented together with the justifications for the methods used, which supported the scientific integrity of the research and the accuracy of the results. In order to ensure data security, the data obtained were stored on a cloud system accessible only to the researchers. This system not only prevented data loss but also prevented unauthorized access to the data. In addition, anonymization of qualitative data and hiding personal information in direct quotations in the text were applied as a basic measure to ensure data confidentiality. This research was conducted within the scope of a national project and ethical processes were meticulously applied. Written consent was obtained from the participants confirming that they voluntarily participated in the study and that they would not experience any ethical problems. The survey used in the study did not carry any political, cultural, or ethically sensitive content.

#### Ethical commitment statement of the study.

The corresponding author undertakes that this study has followed scientific, ethical, and citation rules; no falsification has been made on the collected data, The Journal has no responsibility for any ethical violations that may be encountered, all responsibility belongs to the Corresponding Author, and this study has not been sent to any other academic publication environment for evaluation. The research was conducted with the permission of XXX University Human Research Ethics Committee dated 05/09/2022 and numbered 2022/133.

## Findings

### Integration of technology into DBL lesson plans: Stages and methods used by teachers

In the first sub-objective stage of the study, it is aimed to determine at which stage teachers are closer to using technology while integrating DBL activities into lesson plans. In this context, firstly, the participants were asked whether they were able to integrate technology into their lesson plans and 30 of the 43 participants reported that they were able to integrate technology into their lesson plans. The plans of the teachers who reported that they could not integrate technology (f = 13) were examined and it was determined that there was no integration of technology discipline as they stated. All of the teachers (f = 13) reported that they had difficulty in making technology integration and that they needed suggestions because they could develop ideas as instructional technology. On the other hand, the plans of 30 teachers who declared that they integrated technology into their lesson plans were analyzed according to the first criterion of the descriptive analysis framework. It was determined that technology was used only as a tool in 11 of the lesson plans. These lesson plans included activities on digital education platforms (n = 5), digital stories developed by the teachers themselves (f = 2), assessment activities with digital tools (f = 2), watching videos (f = 1), and doing research on the internet (f = 1). For example, using the Kahoot application as a measurement and evaluation activity, presenting a self-developed digital story to students to create a problem context, or calculating the carbon footprint using a web application were the applications where technology was evaluated only as a tool. In this context, it was determined that technology integration was made in only 19 lesson plans. After the data was collected from the teachers, the data on the frequency of integrating technology and the stages they preferred in this process are given in Table 1.

As presented in Table 3, teachers frequently integrated the discipline of technology at the stage of prototyping and testing (f = 11), communication (f = 11), and developing possible solutions (f = 8). While integrating technology into DBL activities, teachers frequently focused on digital story design (f = 5), artificial intelligence and robotics applications (f = 4), and drawing 3D designs (f = 3). Teachers also integrated technology through applications such as digital poster design (f = 2), interactive games and simulations (f = 2) interactive research (f = 2), or web page design (f = 1). It was found that technology effectively supported the transformation of theoretical knowledge into practice in lesson plans (f = 19) and that technology clearly served the purpose of the activity (f = 19). Teachers used technology interactively that was both innovative and deepened students’ learning processes (f = 16). It was determined that teachers used technology mostly to produce prototypes (f = 11) and partly in processes other than prototypes (f = 8). For example, a teacher planned an activity in which students would design a product using a block-based programming tool. In this activity, students are expected to design a “smart waste container”. This process is considered as ensuring technology integration during the prototyping and testing phases. In addition, the integration of topics such as students’ familiarization with the interface and features of the programming tool, object recognition methods with artificial intelligence, and robot arm working principles enriched the technology integration.

**Table 3:** Findings on how technology integration is provided in teachers’ lesson plans.

Criterion	Content		f
Technology integration in the stages of the DBL process	Identifying the Problem		
	Development of Possible Solutions (f = 8)	Interactive gaming and simulation	4
		Robotics applications	2
		Interactive research	2
	Selection of the Most Suitable Solution		
	Prototyping and Testing (f = 6)	Drawing of 3D designs (Tinkercad)	4
		Robotics applications	2
	Communication (f = 7)	Digital story	4
		Digital poster	2
		Web page design	1
The effectiveness of technology in transforming theoretical knowledge into practice	Technology is ineffective in transforming theoretical knowledge into practice.		
	Technology turns theoretical knowledge into practice to a limited extent.		
	Technology effectively supports the translation of theoretical knowledge into practice.		19
The alignment of technology with the overall objective of the activity	The technology is not directly linked to the purpose of the event.		
	The technology is partially aligned with the purpose of the event but makes a limited contribution.		
	The technology clearly serves the purpose of the event.		19
Innovative and interactive use of technology	The technology is not innovative but remains in passive use.		
	The technology is innovative, but the level of interaction is limited.		
	Technology is innovative and used in interaction with students.		
	Technology is used in an interactive way that is both innovative and deepens the students’ learning process.		16
Physical or digital prototyping	No use of prototypes.		8
	Prototypes are used to a limited extent.		11

Similarly, another teacher explained that she integrated technology in the introductory phase of the lesson to identify students’ prior knowledge and to support their mini-research process as follows: *“Technology integration was used in the introductory phase of the lesson to identify prior knowledge and to support students’ mini-exploration process.” Although the teachers planned DBL activities based on technology integration, they also utilized technology in the process to support teaching.”* For example, some teachers aimed to increase students’ interest in the subject by showing videos on the smart board in the introduction part of their lessons. For example, a teacher defined the problem of seed shortage by showing a video about an ancestor seed and explained the process as follows:

*“We showed videos on the smart board to the students about what is an ancestor seed and news about seed shortage. We also carried out mini research on this topic by discussing that plants are also alive and how they continue their generations.”* On the other hand, a similar teacher used Phet simulations to support students’ learning processes. In order for the students to gain the necessary acquisitions for the problem situation, two different simulations on force and motion were introduced to the students. Then, they were expected to determine and test the variables and prepare an experimental setup through these simulations. This activity provides students with the opportunity to develop their problem-solving skills through technology and shows that the discipline of technology is integrated into the development of possible solutions.

In some lesson plans, prototype designs were used to develop students’ creativity. For example, one teacher planned for students to prepare advertisements for their products using the Powtoon application and integrated technology with digital story design in this process. This shows that technology was effectively integrated in all stages of the activity template, especially in the communication stage. It also encouraged students to work on real-world problems by giving them the task of planning the financial gain of a sustainable recycling facility and how to advertise this gain on the website. Another teacher had the students model their designs in three dimensions using the Tinkercad application. In this process, students first drew prototypes of their designs and planned how they would realize these designs step by step. Then, these plans were transformed into three-dimensional models in the Tinkercad program. This activity includes practical applications of technology and engineering components in STEM education and offers students the opportunity to produce solutions for real-world problems.

### Impact of technology integration in DBL activities on teachers’ professional development

Another sub-objective of our research is to evaluate teachers’ thoughts about technology integration in the engineering design process and the effects of this integration on their professional development. The data obtained in this context are given in Table 4.

**Table 4:** The effect of teachers’ technology integration processes during DBL activities on their professional development status.

Technology integration in the engineering design process	Category	n	%
Direction of effect	It had a positive effect.	29	69.00
	It had a largely positive effect.	13	31.00
Effect on lesson planning skills	It has improved it enormously.	8	19.04
	Improved.	33	78.57
	Not affected.	1	2.38
Effect on technology literacy skills	It has improved it enormously.	9	21.42
	Improved.	31	73.80
	Didn’t change.	2	4.76
Effect on their motivation	Greatly increased my motivation.	9	21.42
	Increased my motivation.	30	71.42
	No effect on my motivation.	2	4.76
	Decreased my motivation	1	2.38

When Table 4 is analyzed, it is seen that all of the teachers (100%) stated that technology integration had/could have a positive effect. 69% of them rated this situation as a positive effect and 31% of them rated it as a positive effect to a great extent. These high rates show that teachers have developed a positive perception due to the innovations and conveniences brought by technology to educational processes. This positive effect on teachers was also observed in the themes obtained from the interviews. For example, ST17 reflected on the positive aspect of this process in terms of technology integration in his professional development by saying *“After the feedback, I made changes in the in-class activities and project assignments for the use of technology, and I will try to apply it in my future studies and plans”*. ST17’s statement reveals that technology offers opportunities for continuous improvement and self-renewal and that teachers have the chance to improve their pedagogical methods. Similarly, ST21 gave clues about the future reflections on the use of technology by stating that “I have observed which applications students can use more fondly in the activities and I think this will be effective in shaping lesson plans”. T21’s observations provide valuable insights into how the use of technology can affect lesson planning by increasing student interest and participation. ST37 stated that he would give importance to “...technology integration in his new lesson plans...”, which emphasizes the importance of technology taking a more active role in teaching processes and shows that teachers show a positive orientation in this direction. When the effect of technology integration on teachers’ lesson planning skills was evaluated, 19% of the teachers reported that there was a “very great improvement” and 79% reported that there was “improvement”. Only a group of 2% reported that the integration had ‘no effect’ on their lesson planning skills. This suggests that technology integration helps teachers with time management, planning lesson flow, and creating diversified learning activities. In addition, the fact that technology-facilitated access to course materials and provided the opportunity to benefit from various resources may have enabled teachers to plan their lessons more effectively. In terms of technology literacy skills, 21 percent of the teachers reported that they had ‘greatly improved’ and 74 percent reported that they had “improved”. Only 5% reported that the integration did not “make a difference” to their technology literacy skills. The improvement in technology literacy skills (21% very much improved and 74% improved) can be explained by the fact that teachers have become more familiar with technological tools and have improved themselves in this process. The necessity to use ever-changing technological tools may have encouraged teachers to adapt to these tools and increase their technological literacy skills. Finally, when the effect of technology integration on motivation was analyzed, 21% of the participants stated that there was a “significant increase”, 71% stated that there was an “increase”, 5% stated that there was “no effect” and 2% stated that there was a “decrease in motivation”. The new teaching methods and tools offered by technology may have enabled teachers to present their lessons more creatively and this may have increased their motivation. Especially few teachers who stated that it had no effect on motivation may not have felt a change in their motivation due to the difficulties of using technology or not getting enough support.

### Teachers’ perspectives on the impact of technology integration in DBL activities on students’ skill development

During the research process, the emotional reactions of teachers to the integration of technology into DBL activities were also analyzed based on the data collected through interviews. It was observed that positive feedback was predominant. The positive nature of this feedback was a matter of curiosity in terms of student-teacher interaction and the student feedback on technology integration into the DBL process was analyzed from the teacher’s perspective. The data obtained are given in Table 5.

**Table 5:** Evaluation distribution for student participation.

Assessment Category	Frequency (%)
Positive	24 (%46.15)
Very positive	26 (%50.00)
Neutral	2 (%3.85)

The data shows that the vast majority of the participants (96.15%) think that they have experienced or think that technology integration has had/will have a positive or very positive impact on student engagement. The “favorable” category of assessment was seen by 46.15%, while the “very favorable” category was more prevalent at 50%. In contrast, only 3.85 percent gave a “Neutral” assessment. Another research question aims to examine which student skills are developed through technology integration in the DBL process. In this context, the data obtained from the analysis of the participants’ responses are presented in Table 6.

**Table 6:** Student skills thought to be improved by the use of technology in the DBL process.

Skills	N	%
Critical thinking	20	15.75
Creativity	30	23.62
Co-operation	22	17.32
Communication	21	16.54
Problem-solving	32	25.20
Other	2	1.57

The results of the analyses show that problem-solving (25.20%) is the skill that is thought to be developed the most in students with technology integration. This is followed by creativity (23.62%), cooperation (17.32%), and communication (16.54%) skills. Critical thinking skills showed a significant improvement with 15.75%. The ‘other’ category was mentioned by only 1.57% of the participants. These results show that technology integration contributes to the development of both cognitive and social skills of students. Especially the high rates of problem-solving and creativity skills support the effective role of technology integration in learning environments. The development of social skills such as communication and cooperation encourage students to take active roles in group work and interdisciplinary projects.

As a result of the analyses, it was seen that most of the teachers stated that technology integration had positive effects on students. This inference is also seen in both direct statements and indirect comments of the teachers. For example, ST10 interpreted the effect of technology integration into the DBL process on students as *“it was positive”*, while ST16 expressed this situation as *“...in general, since the students’ use of technology was good, their feedback was positive”*. These statements show that the integration of technology into educational processes not only enriches learning environments but also positively affects the interaction between teachers and students. ST42 explained the positive aspects of technology integration with examples by stating that students *“...adopt the activities they do too much and compete among themselves to do better...”.* In addition, this situation provides a concrete example that technology integration has an encouraging effect on student motivation and participation and makes the learning process more interactive by creating a healthy competition environment among students. Similarly, ST40 elaborated on the positive reactions of the students by stating that *“...they always asked for this kind of planning, and they saw themselves as more effective and prone to understanding...”.* These observations of the teachers show that the use of technology in education increases students’ interest in learning processes. As a result, it was observed that the emotional reactions of the teachers to the integration of technology into the DBL process were predominantly positive.

On the other hand, some teachers stated that the negative situations they encountered also affected the process. While ST38 explained the ‘time’ problem as “...they could not concentrate because of insufficient time”, ST35 elaborated the same situation as “...they said they would go home and develop it a little more, they needed more time”. These statements show how important it is to provide sufficient time for technology integration to be effective. Time constraints may prevent students from fully exploring the technology tools and displaying their creativity by using these tools. Another problem is ‘the problem of spending time efficiently due to problems such as tool, internet and board breakdown’ mentioned by S36. The problems experienced affected the positive/negative status of technology integration into the DBL process. The same problem was explained by ST8 as *“...students were interested in the use of technology, technical insufficiency of internet and not having a computer/tablet for each student made the process difficult”*. These statements show that the positive emotional state of technology integration is directly related to the reliability and continuity of the tools used. Similarly, ST41, who experienced uncertainty, attributed the reason for this situation to the fact that *“...while the use of technology attracted students to the lesson in general, some students felt inadequate and stayed further away...”.* Therefore, he stated that students should *“...have technological competence beforehand in order not to fall behind the lesson...”.* As a result, teachers stated that factors such as ‘time’ constraints and technical problems prevented students from making full use of technology tools and revealing their creative potential.

One of the most prominent reasons for the positive effects of technology integration into the DBL process on students was the theme of ‘attracting interest’. For example, ST12 expressed this situation through a software tool as ‘The Web 2.0 tools that they will use to transform into a digital story attracted their interest’ and stated that this situation also increased the motivation of the students. In particular, the use of such innovative technologies may have enabled students to actively participate in the learning process and attracted their interest due to the novelty effect. Similarly, ST18 stated that *“students’ interest and desire in the lesson increased”* and associated this situation with cooperation by saying *“...they were very willingly involved in the learning process by cooperating with me”*. ST8 associated the theme of interest directly with the use of technology by saying *“Students were interested in the use of technology”*. This statement shows how technology integration strengthens interaction and collaboration in the classroom. On the other hand, ST13 emphasized that new technologies attracted students’ interest. For example, the process of printing a propeller model on a 3D printer was met with great admiration by the students. The fact that students described the use of such modern technologies in the school environment as “incredible” shows how technology triggers student curiosity and interest. Such innovative applications can increase students’ natural interest in science and technology and enable them to participate more actively in the learning process. To summarize; the positive effects of technology integration into the DBL process on students were particularly concentrated under the theme of “attracting interest”.

According to the teachers’ views, another factor in the positivity of technology integration into the DBL process is that technology makes the lessons “fun/enjoyable” and “motivating”. This theme is encountered in ST6‘s direct statement as *“It is more fun and motivating...”* and in ST42’s comments as *“...they felt more excited, fun and motivated”.* ST12 explained this situation by saying *“...they had higher motivation”*. ST13 stated that the students expressed that the lessons were “fun” because they learned new tools. These statements clearly show how technology integration improves not only the process of knowledge acquisition but also students’ emotional commitment to the lessons and the overall classroom atmosphere. On the other hand, ST20 mentioned the integration of technology into the DBL process as *“the students enjoyed it...”*, while ST21 mentioned the positive aspect by saying *“...they did it with pleasure and the students who did well in using the application made more creative designs”.* The fact that students had the opportunity to express themselves through technological tools also improved their design skills. ST29 drew a general framework by stating that the students *“enjoyed using technology, they were enthusiastic in the learning-teaching process and the use of technology in lessons was beneficial”.* This interest of the students in technology enabled them to participate more actively in the course materials and enriched the learning process. ST17 stated that he observed that *“...their motivation for studying increased...”* because they discovered the existing technological tools and software. ST23, on the other hand, justified this situation by saying *“...they are more motivated in the technology part”* and *“...since we live in the internet age, students are better than us in applying and adapting to technology”.* These statements show the positive effects of technology integration on student motivation and the attractiveness of lessons, which contributed to the improvement of educational processes by making learning environments more interactive and participatory. In conclusion, according to the teachers’ statements, technology integration in the DBL process increased students’ motivation and classroom engagement by making the lessons more fun and motivating. In this process, it is seen that teachers also mentioned some of the skills that the use of technology provides to students. Some of these skills also include 21st-century competencies. For example, ST7 stated that technology integration into the DBL process *“provides students with better critical thinking, problem-solving...”* and emphasized that this situation supports “... permanent learning...” in students. This statement shows that technology plays an important role in developing students’ mental skills and retaining the learned information in long-term memory. ST21 explained the contribution of the process to the students with examples and associated it with creative thinking as *“... students who were good at using the application made more creative designs”.* This situation constitutes a concrete example of how the use of technology triggers students’ creative thinking processes. On the other hand, ST34 inferred that the process *“increased students’ problem-solving skills and self-confidence and expanded their creativity...”* and also stated that it *“...increased their focusing time...”.* Similarly, ST42 stated that the students’ “technology literacy skills” improved and attributed this to the increase in their participation in the lesson. This statement indicates that the use of technology increases students’ interest and participation in the lesson and improves their ability to use technological tools more effectively. As a result, teachers emphasized that technology integration with DBL had positive effects on students’ critical thinking, creativity, and technology literacy skills.

## Discussion and conclusion

### Results of stages and methods of technology integration in DBL lesson plans by teachers

In the current study, teachers first participated in face-to-face training, followed by an 8-week mentoring process and online training. Teachers had mentors whom they could consult while preparing their lesson plans, and mentors also provided feedback on the initial versions of the lesson plans. In such a process, it was found that some teachers did not integrate the technology discipline into DBL activities, while others who attempted to do so not only to facilitate teaching but also to teach in new ways. Half of the teachers moved beyond the basic use of instructional technology in DBL activities by employing technology as a tool for problem-solving in design processes and to enhance student engagement, in alignment with the nature of the STEM approach [57]. This result of the study shows similarities and differences with the literature in various aspects and points to important issues for future studies. How technology should be integrated into the STEM approach is considered a challenging issue for teachers, to the extent that it is recognized as a “mysterious piece” [66,105]. On the other hand, the literature on this subject includes findings that teachers in STEM applications can use technologies mostly as information consumers in a productive manner (searching for information on the internet, summarizing, and making presentations) and less productively as information developers [33]. In our study, the fact that teachers participating in the hybrid mentoring process are attempting to integrate technology into their DBL lesson plans reveals the benefit of using a mentoring approach to overcome the “difficulty of integrating technology” in the literature on technology integration. However, the fact that some teachers did not attempt technology integration and that others who believed they integrated technology did so using the instructional tools indicates that future researchers need to focus on different perspectives to overcome the “challenge of integrating the technology discipline” as noted in the literature. According to the literature, teachers avoid technology integration because they are not sufficiently familiar with technological design, programming, electronics, and other tools [57,63]. However, teachers have concerns and uncertainties about integrating technology into STEM activities [66,105]. Accordingly, it is thought that there is still a need for studies that comprehensively investigate individual factors such as technology anxiety, self-efficacy, and resistance to change, as well as structural factors such as time constraints, pressure from the curriculum, and insufficient resources in schools, and contextual factors such as student readiness. Although individual support is provided to teachers, follow-up studies are needed to examine in depth their individual readiness and characteristics in relation to technology.

Half of the teachers integrated teaching technologies that facilitate the DBL learning process into their activities and prepared design-based activities that could solve real-life problems involving technological features. The findings show that teachers integrated technology in the stages of “developing possible solutions,” “prototyping and testing,” and “communication.” This result indicates that teachers tend to use technology in a product-oriented manner in the prototyping and testing, and communication stages, as well as in a process-oriented manner in the stage of developing possible solutions. Teachers did not prepare design tasks that would enable technology integration in the problem definition stage of the DBL process and facilitate that process in terms of technology integration. Therefore, the integration of technology into the solution development process was also limited. However, [58] argues that problems should include technological parameters from the outset so that students are guided towards technology-based solutions. In this context, teachers’ reduction of technology integration to the solution process contradicts the holistic nature of the DBL process. Teachers’ tendency to position technology as an “auxiliary tool” in their pedagogical approaches indicates that [106]’s recommendation for STEM teachers to “think like experts” in integrated disciplines has not yet been sufficiently addressed and that they do not fully understand the role of technology in STEM education, as stated by [44]. Since teachers did not include technology integration in the problem identification stage, it is parallel/expected that technology integration was not included in the selection of the most appropriate solution due to the absence of technology-related criteria in the problem. Teachers who provided integration in the prototyping and testing stages directed the design of the solution to three-dimensional design and robotic application programs. Teachers supported the communication phase of DBL with digital storytelling, poster design, and web page design for the presentation of the products they designed during the design process to integrate technology. This situation indicates that a professional development process structured with a hybrid mentoring model can be effective in transforming teachers’ practices. In particular, the integration of engineering-based digital tools adds depth to the technology dimension of STEM education and strengthens students’ productive roles [80,107]. This finding provides an important insight into the use of technology in the design phase, which has been largely overlooked in literature. No research has been found in the relevant literature that examines teachers’ integration of technology into the DBL process in depth. However, [29] emphasized that integrating technology into STEM activities as well as the research and DBL process creates learning and design opportunities, thereby enabling full integration. Similarly, studies addressing technology integration in STEM education have concluded that applications where technology is integrated in a way that supports student-centered research and design-based instruction, leveraging the nature of technology, serve the purposes of STEM learning and teaching [58,80,92,107–110]. From this perspective, the DBL activities designed by teachers in this study can be evaluated as serving the technology-related objectives of STEM. Indeed, all of the activities in which teachers integrated technology serve to transform theoretical knowledge into practice and fulfill the purpose of technology. Furthermore, it is an expected result that the technologies used by teachers in the DBL process are innovative and that most of them are interactive in terms of deepening students’ learning processes. However, it is noteworthy that there is limited integration in the creation of technological prototypes for solving real-life problems, which is an important element of the DBL process. Considering the recommendation to work like experts in STEM education particularly in the disciplines integrated within the DBL process [106] for effective technology integration, professional development programs’ content can be designed to enable teachers to engage with technology experts and better understand their work processes. Indeed, it is clear that teachers who want to integrate technology into their lessons need the collaboration and support of experts as well as experienced teacher colleagues (Wells & Velde, 2020). Mentoring support contributes to teachers’ professional development in integrating technology into their lessons [111–114].

### Results of impact of technology integration in DBL activities on teachers’ professional development

It has been determined that teachers have experienced the positive effects of technology integration in DBL activities. In the current study, teachers first had the opportunity to improve their own technology skills and then reflected these technologies in their DBL lesson plans. In this context, teachers evaluated that the technologies they learned during the process provided them with opportunities for personal and professional renewal and development, thereby enabling them to improve their pedagogical aspects/professional development. It is known that technology integration strengthens individuals’ digital skills [115] and supports teachers’ professional development [116,117]. Indeed, teachers stated that technology integration in DBL activities improved their competence in designing DBL activities and enabled them to develop in areas such as time management, organizing lesson flow, diversifying teaching, and making teaching motivating, which are factors affecting effective teaching. It is believed that increased access to digital resources and material diversity in the DBL design process contributed to more effective lesson planning. In this regard, the research findings are consistent with studies showing that technology integration supports teachers’ professional development [118]. In the current study, teachers’ ability to easily reach their mentors and receive quick responses to their questions was aimed at supporting this pedagogical development. Indeed, mentees exchanged professional knowledge with their mentors during both the activity design and implementation processes. Following the mentors’ guidance and warnings on which technology or technological design process would be effective in the science subject they had identified, how the process should be carried out, and how the guidelines should be expressed, teachers were encouraged to reflect on the teaching and learning behaviors they had observed. The reflection, evaluation, and support process provided by mentors to teachers is expected to help them identify their teaching strengths and areas for improvement [119,120]. Our research is consistent with this literature. However, digital/technology literacy and pedagogical development are two factors that support each other and affect teachers’ professional development and the effectiveness of their teaching [121,122]). The fact that teachers who developed their knowledge of technology during the mentoring process reported positive developments in their professional development is consistent with this literature. In our study, it was found that the positive outcomes experienced by teachers using new teaching methods and technologies through technology-integrated DBL activities affected their motivation. It is expected that the adequacy of technology-integrated activities designed by teachers will be dependent on their professional knowledge and motivation, which will ultimately improve the quality of teaching [61]. Indeed, in the study, teachers reported that their motivation was negatively affected by the difficulties they encountered while integrating technology into the process, using it in teaching, and applying it, or by their need for more support, albeit in a limited number of cases. In this context, it is recommended that supportive training programs and infrastructure opportunities be strengthened and sustained so that technology integration can contribute more effectively to teachers’ professional development and motivation processes.

### Results regarding teachers’ views on the effects of technology integration into DBL activities on students’ skill development

As discussed in the first and second research questions of this study, technology in the DBL lesson planning and implementation process has a positive impact on teachers’ integration of technology into the DBL process and their professional development. It also affects students’ attitudes, motivation, and tendencies toward designing, implementing, and improving technology-integrated DBL activities. Indeed, in the current study, teachers who integrated and applied technology in the DBL process stated that their students’ participation in the course increased and that it supported the development of their cognitive, affective, and social skills in addition to science learning. Teachers emphasized that technology DBL contributed to the development of their students’ problem-solving, creativity, communication, collaboration, and critical thinking skills in particular. These views of teachers are consistent with the literature. Indeed, DBL activities enable students to learn science while developing their problem-solving skills [123], creative thinking [124,125], collaboration and communication [126], and critical thinking [127] skills, which in turn increases their motivation to learn science [108]. As discussed in the context of the second sub-problem of the study, some teachers stated that technology integration negatively affected the process itself, as well as student motivation and development, due to time constraints and technical infrastructure deficiencies. This situation is related to their professional knowledge and skills regarding technology integration in the DBL process. Although teachers were supported by a hybrid mentoring-based professional development program in the current study, they were unable to overcome such problems effectively. In addition, although most of them believe that integrating technology into the DBL process will support students’ skill acquisition, they have avoided integrating technology into the DBL process, probably to avoid similar situations. Students’ motivation and development affect teachers’ motivation to teach and, consequently, their professional development [128]. Mentoring is effective in making this situation visible [129]. Considering the impact of systematic and continuous mentoring on making teacher development sustainable [130], it is recommended to either sustain mentoring in the long term or structure the mentoring process by taking individual characteristics into account in order to overcome this situation.

### Limitations and suggestions

In this study, it was aimed to examine how science teachers, who were participants of a nationwide project receiving mentoring support for the use of the DBL method in science lessons, realized technology integration in DBL lesson plans and their experiences. It was found that teachers mostly attempted to integrate technology into their DBL activities and most of them integrated technology to facilitate teaching as well as to teach in new ways. Despite mentoring support, they had difficulties in integrating technology into the DBL process. It was found that they had the opinions that technology integration in DBL activities had a significant positive effect on their lesson-planning skills, technology literacy, and motivation. On the other hand, teachers’ inferences about the contribution of the process to students were that technology integration increased student motivation and participation, made learning environments more interactive, and supported skills such as creativity and problem-solving by increasing teacher-student interaction. One limitation of the study is that the finding that technology integration may cause stress and anxiety in some teachers cannot be explained in detail. This situation requires a more in-depth examination of factors such as teachers’ technology literacy levels, professional experiences, and individual perceptions. However, it may not be sufficient to explain the effectiveness of technology integration only with the teacher factor; factors such as the physical and digital infrastructure of learning environments, curricula, and individual differences of students should also be taken into consideration. Although a mentoring process is being carried out to provide individual support to teachers, follow-up studies are needed to thoroughly examine the impact of their individual readiness and characteristics on technology integration. Additionally, comprehensive studies could be conducted to examine how factors such as the technical infrastructure and school climate in the schools where teachers work influence their orientation toward technology integration in DBL activities. It is recommended that professional development programs’ content be prepared to bring teachers together with technology experts to develop their understanding of how to integrate technology into the STEM approach. Sample materials can be prepared to prevent teachers from limiting technology integration in the STEM approach to instructional technology. Materials containing sample lesson plans that demonstrate how technology can be integrated into each stage of the DBL process can support the mentoring process. In addition, improving time management and technical infrastructure will contribute to a more continuous and effective implementation of DBL processes.

## Supporting information

S1 File DBL Lesson Plan Template. Provides a structured template for preparing design-based learning lesson plans. It includes sections for grade level, learning area, objectives, materials, prior knowledge assessment, techniques, and all stages of the engineering design process (problem identification, developing possible solutions, selecting the most appropriate solution, prototyping and testing, and communication).
(DOCX)

## Abbreviations

DBL: design based learning
LMS: learning management system
STEM: science, technology, engineering, mathematics
