# Supplementary material for: Science teachers’ mentoring support experiences when integrating technology in design-based learning STEM activities
Source: PLoS One. 2025 Sep 29;20(9):e0332047. doi: 10.1371/journal.pone.0332047 (PMC12478917; doi:10.1371/journal.pone.0332047)
Supplement: S1 File — Provides a structured template for preparing design-based learning lesson plans. It includes sections for grade level, learning area, objectives, materials, prior knowledge assessment, techniques, and all stages of the engineering design process (problem identification, developing possible solutions, selecting the most appropriate solution, prototyping and testing, and communication). (DOCX) [file pone.0332047.s001.docx]

**Appendix 1. DBL Lesson Plan Template**

| **Grade Level** | *Write which grade level the activity is appropriate for.* |
| --- | --- |
| **Learning Area** | *Indicate which learning area of the science curriculum the activity is related to.* |
| **Topic and Objectives** | *Write which topics and learning outcomes the activity covers.* |

| **Materials** | ***List the materials and resources required for the activity in bullet points.***   - **….** - **….** - **…..** |
| --- | --- |
| **Characteristics of the Learning Environment** | ***Describe the environment in which this activity can be carried out.*** |

| **ASSESSMENT OF PRIOR KNOWLEDGE** | **WHAT PREREQUISITE KNOWLEDGE DO STUDENTS NEED TO HAVE FOR THIS ACTIVITY?** |
| --- | --- |
|  | *What prior knowledge should students have when the lesson plan is implemented?*  *Will their current knowledge and skills be sufficient? (Or will they need to learn a program such as Arduino?)*  Please list the activities you will conduct to identify prior knowledge. |

| **Method Used** | *Design-Based Learning Method* |
| --- | --- |
| **Techniques** | Specify the techniques you will use for mini research and applications. |

1. **Stage: Problem Identification**

| ***Problem context*** | Here, you must describe the real-life context of the engineering design problem.  *The real-life context you established before the engineering design problem can be described through a video, newspaper article, case study, etc. When creating problems, students should pay attention to the real-life context.* |
| --- | --- |
| ***Presentation of the problem situation*** | Write your problem situation here. Do not forget the characteristics that your problem situation should have. |
| ***Problem identification*** | *Students should be given instructions to understand what is required in the problem and to identify the criteria and constraints. They should be asked questions to ensure they understand what is required in the problem.*  Describe the process you plan to carry out in class during the problem identification stage. |
| ***Design activities are carried out in groups. At this stage, students should be divided into groups. When forming groups, pay attention to the rules for group formation.*** | |

1. **Stage: Developing Possible Solutions**

| ***Identifying Needs Related to the Design Problem*** | Describe what students need to know to solve the problem and what you will do. |
| --- | --- |
| ***Conducting Mini Research and Design*** | In this phase, activities planned using inquiry-based research techniques should be detailed to provide the knowledge and skills needed to develop solutions to the problem.  *(Note: You can briefly describe the activities you will carry out here and attach the activity sheets as an appendix at the end of the plan.)*   1. *Activity: …….* 2. *Activity: ……..* |
| ***Development of Individual Solution Proposals*** | After students have acquired the necessary knowledge and skills, they are given time to individually generate solutions to the problem.  *(At this stage, students should not be allowed to interact with each other.)*  Please describe how you plan to implement this phase. |
| ***Groups come together and share their solution proposals*** | *Groups come together to share their individual solutions and develop group solutions.*  Describe how you plan to structure this phase. |

1. **Stage: Selecting the Most Appropriate Solution**

| **Groups' Solution**  **Proposals**  **Evaluate-**  **the** | Group work should continue at this stage. Students are expected to evaluate their solution proposals based on the criteria and constraints.  *You can use techniques such as decision trees and decision matrices for this stage. You can find information about these techniques in the videos [the most suitable video for selecting the solution using decision matrices and decision trees is available at ].*  Please explain your plan here. |
| --- | --- |
| **Selection of the Most Appropriate Solution** | At this stage, students are expected to explain why they believe their chosen solution is the best. They should be encouraged to use concepts such as "trade-offs" in their explanations.  Explain your plan here. |

1. **Stage: Prototyping and Testing**

| **Prototype Development/**  **Solution Development** | *In the engineering design process, the product can be a model, a process design, or a symbolic model of an evidence-based solution (Kelly and Cunningham, 2019).*  *At this stage, groups should be given the opportunity to develop their solutions. Before implementing their chosen solutions, group decisions should be listened to separately and the solution should be implemented after teacher approval.*  Explain your planning process here. |
| --- | --- |
| **Testing** | Students should be asked to present their drawings or models, which should be evaluated using graded scoring keys. This section should explain how testing will be conducted. The rubric to be used for testing should be provided separately or included here. |
| **Improvement** | After testing, groups should be given the opportunity to improve their solutions. They should explain the changes they made and retest. |

1. **Stage: Communication**

| **Presentation of Designs** | In this stage, groups should present their designs and evaluate them in terms of their compliance with the criteria.  Please explain your plan here. |
| --- | --- |
| **Summary** | The teacher should summarize the process by highlighting the knowledge and skills (in line with the learning outcomes) used by students in generating solutions to problems. |

| **Assessment Activities:** |
| --- |
| List here the assessment activities you will conduct throughout the process (excluding the testing of the design). |

| **Attachments:** |
| --- |
| You can provide additional materials such as activity sheets, visuals, or case studies that you will use in each stage of the lesson plan in this section. |
